# Supplementary material for: The interactome of intact mitochondria by cross-linking mass spectrometry provides evidence for coexisting respiratory supercomplexes
Source: Mol Cell Proteomics. 2017 Dec 8;17(2):216–32. doi: 10.1074/mcp.RA117.000470 (PMC5795388; doi:10.1074/mcp.RA117.000470)
Supplement: Supplemental Data [file supp_RA117.000470_133922_0_supp_23978_fzffwf.zip › spectra_annotation/mito_DR_spectra_annotation/21-1-8-1-5-1.pdf]

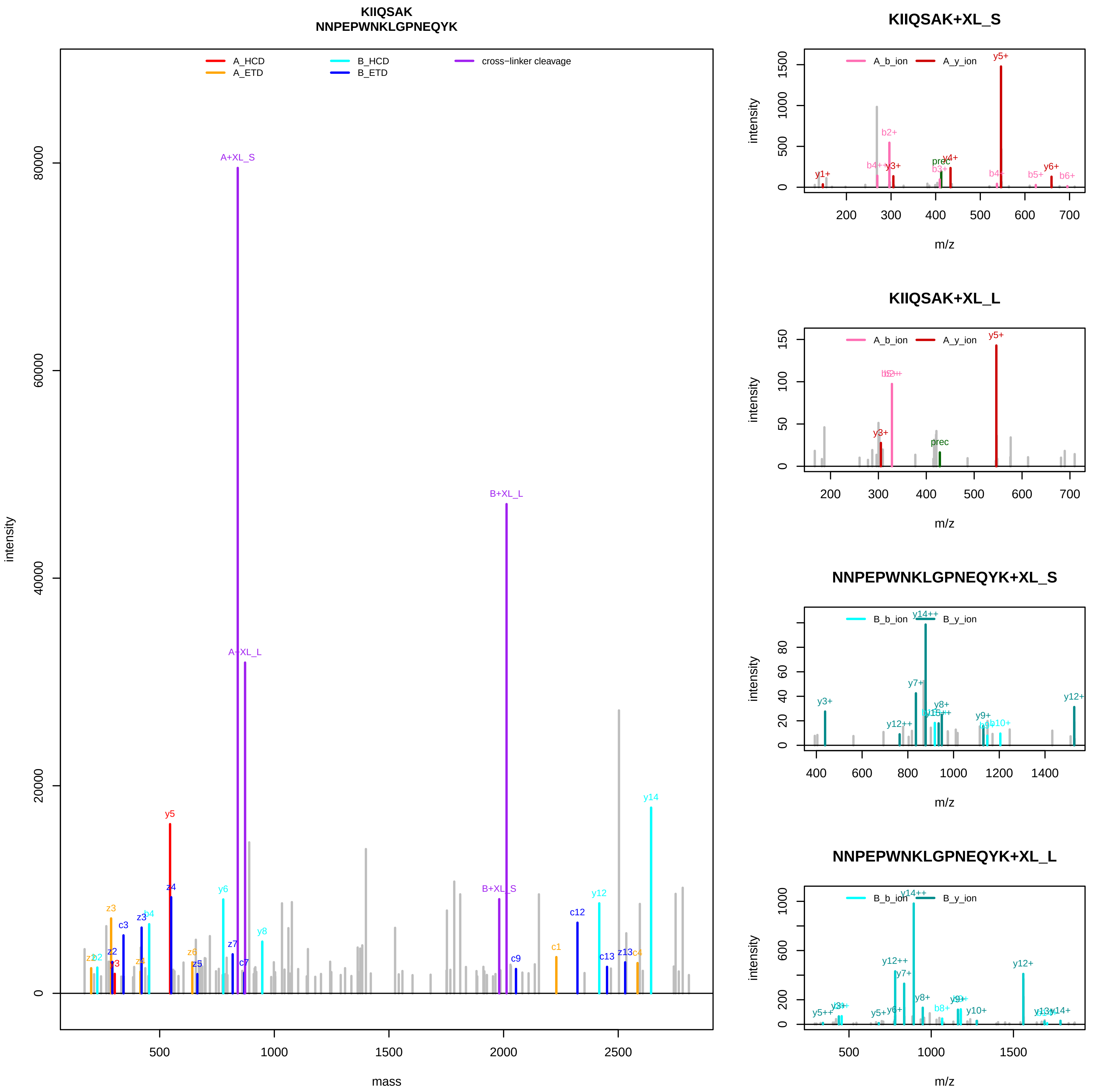
KIIQSAK
NNPEPWNKLGPNEQYK
A_HCD
A_ETD
B_HCD
B_ETD
cross−linker cleavage
intensity
mass
A+XL_S
A+XL_L
B+XL_L
B+XL_S
y5
z4
y6
y8
z7
c7
z5
z6
z3
c3
z2
y3
z4
b4
c9
c1
c12
y12
c13
z13
c4
y14
KIIQSAK+XL_S
KIIQSAK+XL_L
NNPEPWNKLGPNEQYK+XL_S
NNPEPWNKLGPNEQYK+XL_L
A_b_ion
A_y_ion
B_b_ion
B_y_ion
m/z
prec
y1+
b2+
b3+
y3+
y4+
y5+
y6+
b5+
b6+
y7+
y8+
y9+
y10+
y12+
y12++
y14++
y14+
y5++
y5+
y6+
b8+
